# *Enterococcus faecium* NCIMB 10415 administration improves the intestinal health and immunity in neonatal piglets infected by enterotoxigenic *Escherichia coli* K88

**DOI:** 10.1186/s40104-019-0376-z

**Published:** 2019-08-21

**Authors:** Xie Peng, Ru Wang, Liang Hu, Qiang Zhou, Yang Liu, Min Yang, Zhengfeng Fang, Yan Lin, Shengyu Xu, Bin Feng, Jian Li, Xuemei Jiang, Yong Zhuo, Hua Li, De Wu, Lianqiang Che

**Affiliations:** 1 Key Laboratory for Animal Disease-Resistant Nutrition of the Ministry of Education of China, Institute of Animal Nutrition, Sichuan Agricultural University, Chengdu, Sichuan 611130 People’s Republic of China; 2 Animal Husbandry and Veterinary Department, Chengdu Agricultural College, Chengdu, Sichuan 611130 People’s Republic of China

**Keywords:** *Enterococcus faecium*, Enterotoxigenic *Escherichia coli* K88, Gut microbiota, Immunity, Intestine, Neonatal piglets

## Abstract

**Background:**

This study aimed to investigate the effects of oral administration of *Enterococcus faecium* NCIMB 10415 (*E. faecium*) on intestinal development, immunological parameters and gut microbiota of neonatal piglets challenged with enterotoxigenic *Escherichia coli* K88 (ETEC). A total of 96 1-day-old sow-reared piglets were randomly assigned to 2 groups, with 48 piglets in each group. The piglets were from 16 litters (6 piglets each litter), and 3 piglets each litter were allocated to the *E. faecium*-supplemented (PRO) group, while the other 3 piglets were allocated to the control (CON) group. After colostrum intake, piglets in the PRO group were orally administrated with 3 × 10^9^ CFU *E. faecium* per day for a period of one week. On day 8, one piglet per litter from each group was challenged (CON+ETEC, PRO+ETEC) or not (CON-ETEC, PRO-ETEC) with ETEC in a 2 × 2 factorial arrangement of treatments. On day 10 (2 days after challenge), blood and tissue samples were obtained from piglets.

**Results:**

Before ETEC challenge, there were no significant differences for the average daily gain (ADG) and fecal score between the two groups of piglets. After ETEC challenge, the challenged piglets had greater fecal score compared to the non-challenged piglets, whereas *E. faecium* administration was able to decrease the fecal score. Piglets challenged with ETEC had shorter villous height, deeper crypt depth, and reduced number of goblet cells in the jejunum and decreased mRNA abundance of *claudin-1* in the ileum, whereas increased the percentage of lymphocytes, concentrations of IL-1β in the plasma and TNF-α in the ileal mucosa, as well as increased the mRNA abundances of innate immunity-related genes in the ileum tissue. These deleterious effects caused by ETEC were partly alleviated by feeding *E. faecium*. In addition, piglets in PRO-ETEC group had decreased the percentage of CD8^+^ T cells of the peripheral blood when compared to those in CON-ETEC group. Moreover, *E. faecium* administration increased Verrucomicrobia at phylum level and decreased *Bilophila* at genus level.

**Conclusions:**

These results suggest that oral administration of *E. faecium* alleviated the intestinal injury and diarrhea severity of neonatal piglets challenged by ETEC, partly through improving the intestinal microbiota and immune response. This offers a potential strategy of dietary intervention against intestinal impairment by ETEC in neonatal piglets.

**Electronic supplementary material:**

The online version of this article (10.1186/s40104-019-0376-z) contains supplementary material, which is available to authorized users.

## Background

The gastrointestinal tract of neonatal piglets is vulnerable to diarrhea during the early-life period [1]. Before birth, the intestinal tract of fetus has been assumed to be sterile, while the newborns have been colonized by a complicated community of microbiota [2–4]. Dysregulation or imbalance of the neonatal gut microbiota may lead to higher risk of diseases and long-term negative effects on host health [5]. Increasing evidences showed that early microbiota colonization could affect the microbial composition and immunological maturation [6, 7]. Thus, early colonization with beneficial bacteria resulting in the establishment of a stable bacterial ecology may have practical significance to improve the health of neonatal piglets.

Neonatal piglets are often infected with pathogenic bacteria derived from either maternal or environmental source [8]. The infection of ETEC K88 is one of the important causes for diarrhea of neonatal and post-weaning piglets, resulting in lower growth rate and great economic loss in the pig farming [9]. Severity of diarrhea can be up to 50% in suckling piglets [10]. ETEC produces enterotoxins that disturb the gut microbiota and stimulate the loss of water and electrolytes, leading to diarrhea [11, 12]. Numerous studies have showed that probiotics can inhibit the growth of potential pathogens and prevent diarrhea in pigs [13–15]. *Enterococcus faecium* (*E. faecium*) is one of lactic acid bacteria with inhibitory effects against several important enteric pathogens [16]. It has been demonstrated that oral administration of *E. faecium* from birth to weaning had the potency to reduce the diarrhea severity [17]. In addition, *E. faecium* was previously used to improve intestinal microbial balance of pigs [18, 19], modulate the composition of blood lymphocytes [20], and regulate the immunological homeostasis in the intestine [21, 22]. However, under ETEC challenge, the effects of oral administration of *E. faecium* on diarrhea severity, intestinal microbial community structure and immunological parameters in neonatal piglets have rarely been reported.

Therefore, the aim of this study was to determine the hypothesis that oral administration of *E. faecium* could improve the negative effects of ETEC infection on intestinal function, microbiota and immune responses of neonatal piglets.

## Materials and methods

### Animals, diets and treatments

All experimental procedures followed the current law regarding animal protection (Ethic Approval Code: SCAUAC201308–2) and were approved by the Guide for the Care and Use of Laboratory Animals prepared by the Animal Care and Use Committee of Sichuan Agricultural University. Sixteen litters of newborn piglets (10~11 piglets in each litter), derived from sixteen sows with the similar parities (parity 3~4). All the piglets were delivered vaginally at the Giastar Pig Experimental base (Duroc × Landrace × Yorkshire) and allowed the consumption of colostrum for 24 h. Afterwards, a total of 96 piglets (6 piglets each litter) with an initial BW of 1.72 kg (SEM 0.05) were selected, and 3 piglets each litter were allocated to the PRO group, while the other 3 piglets were allocated to the control (CON) group. All the piglets were sow-reared. Piglets in the PRO group were orally administered 3 × 10^9^ CFU *Enterococcus faecium* NCIMB 10415 per day, dissolved in 9 mL of physiological saline and 3 mL of solution was given by using a 10 mL syringe without the needle at 08:00, 14:00 and 20:00 h. The counts of viable probiotic bacteria in the *E. faecium*-containing supplements were verified via cultural method using a selective medium [23]. Piglets in the CON group were orally administered with the same volume of physiological saline. No antibiotics were given to the animals throughout the trial for prophylactic or therapeutic reasons. The oral administration lasted 7 days from the age of 2 to 8 days. One week after administration, the body weight was measured and average daily weight gain (ADG) was calculated [24, 25]. The fecal scores were recorded according to the following criteria: 0, normal; 1, pasty; 2, semiliquid; and 3, liquid [26].

At 08:00 h on day 8, one healthy male piglet with BW closest to the average BW of each litter was selected from each group (*n* = 16). Piglets were checked daily for clinical signs (i.e., diarrhea, dehydration, and apathy) to evaluate their health status before challenging with ETEC. Eight of piglets in each group were orally administered with 80 mL of sterilised Luria broth as the unchallenged group, while another 8 piglets were administered with an equivalent amount of Luria Broth containing 10^9^ CFU/mL ETEC (serotype O149:K91:K88ac; China Veterinary Culture Collection Center) as the challenge group, as previously reported [27]. Therefore, four groups of piglets were created and studied: CON-ETEC; CON+ETEC; PRO-ETEC; PRO+ETEC (*n* = 8). The experimental design is detailed in Additional file 1: Figure S1. To prevent cross-contamination between groups, high hygienic standards were maintained at all times, including the change of disposable boots, coverall and gloves between rooms, and the unchallenged and ETEC-challenged piglets were housed in their respective nursing cages (0.8 m × 0.7 m × 0.4 m) by separate sanitary rooms. Room temperature was maintained at approximately 30 °C, and the humidity was controlled between 50% and 60% [28].

The fecal scores were recorded at 4, 8, 12, 16, 20 and 24 h after ETEC challenge. During the challenge study, piglets were bottle-fed individually with formula milk every 3 h between 06:00 and 24:00 daily. The formula milk was formulated according to our previous study [29]. The liquid formula milk was prepared by mixing 1 kg of formula powder (DM 87.5%) with 4 L of water, in which nutrients composition and levels were similar as sow milk [30]. All piglets had free access to drinking water.

### Bacterial strain

The ETEC K88 strain (serotype O149:K91:K88 ac; China Veterinary Culture Collection Center) was grown in Luria broth medium containing 1% tryptone, 0.5% yeast extract, and 1% NaCl, pH 7.0. Tryptone and yeast extract were from Oxoid (Basingstoke, England). After overnight incubation at 37 °C with shaking, bacteria were diluted 1:100 in fresh Luria broth. Following incubation, the bacterial cells were harvested by centrifugation at 3,000×*g* for 10 min at 4 °C, washed in sterile physiological saline, and resuspended in saline. Bacteria grown to mid-log phase (about 0.5 OD600) were used for the challenge experiment. Bacterial concentration was determined by densitometry and confirmed by serial dilution followed by viable plate counts on Luria broth agar.

### Blood sampling

At 08:00 h after 2 days of ETEC challenge, blood samples were collected from the anterior vena cava after an overnight fast. Blood samples of 2 mL were injected into Eppendorf tubes containing sodium heparin for the examination of routine blood and flow cytometry analysis within 2 h after collection. Blood samples of 8 mL were contained in heparinized tubes, followed by 3,000×*g* at 4 °C for 15 min, plasma was separated and then immediately stored at − 80 °C for later analysis.

### Tissue sample collection

After blood sampling on day 10 (2 days after challenge), piglets in the ETEC challenge study (*n* = 8) were sedated with an intravenous injection of pentobarbital sodium (10 mg/kg BW) and euthanized with an intramuscular injection of pentobarbital sodium (15 mg/kg BW) followed by a subsequent exsanguination protocol approved by the Sichuan Agricultural University Animal Care Advisory Committee. After the abdomen was exposed, jejunal sample of approximately 2 cm in length was stored in 4% paraformaldehyde solution for histological measurements. Ileal segments (6 cm in length) were opened longitudinally, and washed with ice-cold PBS to remove digesta. Mucosa was gently scraped with a sterile glass microscope slide at 4 °C, rapidly frozen in liquid N_2_ and stored at − 80 °C for further analysis of inflammatory cytokines. Another ileum (2 cm in length) tissue samples were collected, snap-frozen and stored at − 80 °C for the analysis of mRNA expression. Approximately 10 g of colonic digesta from each piglet was dispensed into two sterilized 5-mL centrifuge tubes, and immediately frozen at − 80 °C for later analysis of bacterial 16S rRNA and short chain fatty acids (SCFAs).

### Routine blood examination and lymphocyte subtype analysis

Routine blood examination included lymphocytes, neutrophils, intermediate cells, red blood cells, haematocrit, mean corpuscular volume, platelets, thrombocytocrit, and white blood cells. These parameters were analyzed using an automatic blood analyzer (Advia 120, Bayer HealthCare, Tarrytown, NY). Lymphocyte subtype was measured by a FACS Calibur flow cytometer (Becton, Dickinson and Company, San Jose, CA). Briefly, mouse anti-porcine CD3, mouse anti-porcine CD4, and mouse anti-porcine CD8 (Southern Biotechnology Associates, Birmingham, AL, USA) were added into 100 μL of blood in a 12 mm × 75 mm tube. The tube was gently mixed and incubated for 30 min in the dark at room temperature, then added 1 mL of RBC lysing solution (BD Biosciences, USA) and incubated for another 10 min. The cocktail was centrifuged at 500×*g* for 5 min, then re-suspended with PBS and detected by flow cytometer. The percentage of CD3^+^, CD4^+^, and CD8^+^ lymphocytes were determined by CellQuest software program (BD Biosciences, USA).

### Plasma and ileal cytokines analysis

Before measurement, approximately 0.1 g of frozen ileal mucosa was homogenized in 10 volumes (1:10, *w*/*v*) of ice-cold physiological saline by using ultrasonic cell disruption system (Scientz-IID, Scientz, Ningbo, China) at 4 °C, and then centrifuged at 4,500×*g* for 15 min at 4 °C. The ileal supernatant and plasma were used to detect the concentrations of interleukin 1β (IL-1β), IL-6, and tumor necrosis factor α (TNF-α) with commercial ELISA kits (Beijing 4A Biotech Co., Ltd., Beijing, China) according to the manufacturer’s instructions.

### Small intestinal morphology and goblet cell counting

The histomorphology and the count of goblet cells in the jejunum were determined according to our previous study [30]. Briefly, each tissue sample was used to prepare five slides and each slide had three sections (5 μm thickness), which were stained with hematoxylin and eosin for intestinal morphology analysis of 20 intact well-oriented crypt–villus units each section (Scion Image software, Version 4.02, 2004). Periodic Acid Schiff and Alcian Blue (PAS-AB) were used for counting goblet cells. The number of positively stained goblet cells was measured (NIS-Elements BR 2.3; Nikon France SAS), and the values obtained from 10 villi by each small-intestinal segment were averaged.

### Total RNA extraction and real-time reverse transcription PCR

Total RNA was extracted from frozen ileal samples using Trizol reagent (TaKaRa Biotechnology, Dalian, China) according to the manufacturer’s instructions. The quality of the RNA was determined by electrophoresis on 1.0% agarose gel, and the purity of RNA was assessed by evaluating the OD260:OD280 ratio using a nucleic acid analyzer (Beckman DU-800; Beckman Coulter, Inc., Brea, CA) [31]. Both genomic DNA removal and reverse transcription were performed using PrimeScript RT reagent kit with gDNA eraser (TaKaRa Biotechnology) according to the manufacturer’s guidelines. Real-time PCR was performed using SYBR Premix Ex Taq (Tli RNaseH Plus) qPCR kit (TaKaRa Biotechnology Dalian Co., Ltd., Dalian, China) with ABI-7900HT Fast Real-Time PCR System (Applied Biosystems, Foster City, CA, USA). The PCR reaction consisted of 5.0 μL SYBR Premix Ex Taq (2×), 0.4 μL forward primer (10 μmol/L), 0.4 μL reverse primer (10 μmol/L), 0.2 μL ROX reference dye (50×), 1.0 μL cDNA, and 3.0 μL double-distilled water in a total volume of 10 μL. The PCR procedure was as follows: pre-denaturating at 95 °C for 30 s, 40 cycles of denaturation at 95 °C for 5 s, annealing at 60 °C for 34 s, and extension at 95 °C for 15 s and a cycle of final extension at 72 °C for 6 min. At the end of amplifcation, melting curve analysis was performed to verify specifc amplifcations. *β-actin* was used as an internal reference gene to normalize the expression of target genes according to the 2^-ΔΔCt^ method described by Livak and Schmittgen [32], where ΔΔCt = (Ct_target_ − Ct_β-actin_)_treatment_ − (Ct_target_ − Ct_β-actin_)_control_. The mRNA level of each target gene for CON-ETEC group was set to 1.0. All samples were run in triplicate, and the primers are shown in Table 1.

**Table 1:** Primer sequences of target and reference genes

Gene	Primer sequence (5'→3')	Product, bp	GenBank accession
*TLR-9*	Forward: AATCCAGTCGGAGATGTTTGCT	79	AY859728
	Reverse: GACCGCCTGGGAGATGCT		
*TLR-2*	Forward: TCGAAAAGAGCCAGAAAACCAT	58	NM213761
	Reverse: CTTGCACCACTCGCTCTTCA		
*TLR-4*	Forward: AGAAAATATGGCAGAGGTGAAAGC	64	GQ304754
	Reverse: CTTCGTCCTGGCTGGAGTAGA		
*MyD88*	Forward: GTGCCGTCGGATGGTAGTG	65	NM001099923
	Reverse: TCTGGAAGTCACATTCCTTGCTT		
*TRAF-6*	Forward: GCTGCATCTATGGCATTTGAAG	70	AJ606305.1
	Reverse: CCACAGATAACATTTGCCAAAGG		
*NF-κB*	Forward: TGCTGGACCCAAGGACATG	60	AK348766.1
	Reverse: CTCCCTTCTGCAACAACACGTA		
*IL-6*	Forward: GATGCTTCCAATCTGGGTTCA	62	M80258.1
	Reverse: CACAAGACCGGTGGTGATTCT		
*Claudin-1*	Forward: TCTTAGTTGCCACAGCATGG	106	NM001244539
	Reverse: CCAGTGAAGAGAGCCTGACC		
*Occludin*	Forward: TTCATTGCTGCATTGGTGAT	113	NM001163647
	Reverse: ACCATCACACCCAGGATAGC		
*ZO-1*	Forward: CCGCCTCCTGAGTTTGATAG	97	AJ318101
	Reverse: CAGCTTTAGGCACTGTGCTG		
*β-actin*	Forward: GGCGCCCAGCACGAT	66	DQ845171.1
	Reverse: CCGATCCACACGGAGTACTTG		

*TLR* Toll-like receptor, *MyD88* myeloid differentiation factor 88, *TRAF-6* TNF receptor-associated factor 6, *NF-κB* nuclear transcription factor kappa B, *IL* interleukin, *ZO-1* zonula occludens-1

### 16S rRNA gene sequencing

The total genomic DNA was extracted from a random subset of colonic digesta (*n* = 6) using the QIAamp DNA stool Mini Kit (Qiagen, GmbH Hilden, Germany) according to the manufacturer’s protocols. The concentration and purity of the extracted genomic DNA were measured using a NanoDrop ND-1000 Spectrophotometer (NanoDrop Technologies Inc., Wilmington, DE, USA). The integrity of the extracted genomic DNA was determined by electrophoresis on 1% (*w*/*v*) agarose gels [33]. Extracted fecal DNA samples were sent to Novogene Bioinformatics Technology (Beijing, China) to perform amplicon pyrosequencing on the Illumina HiSeq PE250 platforms. The V4 hypervariable region of the 16S rRNA gene was amplified by PCR with primers 515F (5′-GTGCCAGCMGCCGCGGTAA-3′) and 806R (5′-GGACTACHVGGGTWTCTAAT-3′). The effective tags were mapped to OTUs using Uparse v7.0.1001 at 97% sequence similarity. Representative sequences for each OTU were selected. The Ribosomal Database Project (RDP) classifier Version 2.2 was used to assign a taxonomic rank to each representative sequence. The relative abundance of each OTU was examined at different taxonomic levels. Diversity within communities (Alpha diversity) calculations and taxonomic community assessments were performed by Qiime 1.7.0. Principal coordinates analysis plots were produced using unweighted UniFrac metrics.

### SCFAs analysis

The SCFAs concentrations in the colonic digesta were measured using a gas chromatographic method as described by Chen et al. [34]. Briefly, digesta samples (1 g) were thawed and suspended in 2 mL of distilled water in a screw-capped tube. After being vortexed, the suspension liquid was centrifuged (12,000×*g*) at 4 °C for 10 min. The supernatant (2 mL) was transferred into Eppendorf tubes and mixed with 0.2 mL metaphosphoric acid. The tubes were placed at 4 °C for 30 min and then centrifuged (12,000×*g*) again at 4 °C for 10 min. Aliquot of the supernatant (1 μL) was analyzed using a Varian CP-3800 gas chromatograph (Agilent Technologies, Santa Clara, CA, USA) equipped with a flame ionization detector and a polyethene glycol packed column (0.32 mm internal diameter, 30 m length and 0.25 μm film thickness). SCFAs were quantified using external standard curves from 0.5 to 100 μmol/mL of the respective authentic organic acids (Fluka, Switzerland) [35].

### Statistical analysis

All data were expressed as means with their standard errors. Growth performance and diarrhea score of piglets before ETEC challenge was analyzed using the unpaired *t* test. The data in the challenge study were analyzed as a 2 × 2 factorial with the general linear model procedures of the Statistical Analysis Package. The model factors included the effects of *E. faecium* administration (with or without *E. faecium* in diets), ETEC infection (ETEC unchallenged or challenged), and their interaction. Data were analyzed using SAS (version 9.4; SAS Inst. Inc., Cary, NC, USA). For analysis of intestinal microbiota, data of relative abundance at phylum and genus levels were log-transformed before statistical analysis. Significant differences were set at *P* ≤ 0.05, whereas 0.05 < *P* <  0.10 was considered a tendency.

## Results

### Growth performance

As shown in Table 2, there were no significant differences (*P* > 0.05) between the two groups of piglets for their ADG and fecal score from the age of 2 to 8 days.

**Table 2:** Effect of *E. faecium* on growth performance and fecal score of piglets

Items	CON	PRO	*P*-value
Initial BW, kg	1.70 ± 0.05	1.73 ± 0.05	0.70
Final BW, kg	3.09 ± 0.07	3.19 ± 0.07	0.37
ADG, g/d	199 ± 7.00	209 ± 8.00	0.37
Fecal score^1^	0.07 ± 0.03	0.04 ± 0.02	0.27

Data are presented as means ± SE (*n* = 16; pen was used as experimental unit)

*CON* control group, *PRO Enterococcus faecium*-supplemented group, *BW* body weight, *ADG* average daily gain

^1^Fecal score = (Sum of the fecal score over the period)/(experiment days)

### Fecal score

**Fig. 1:**
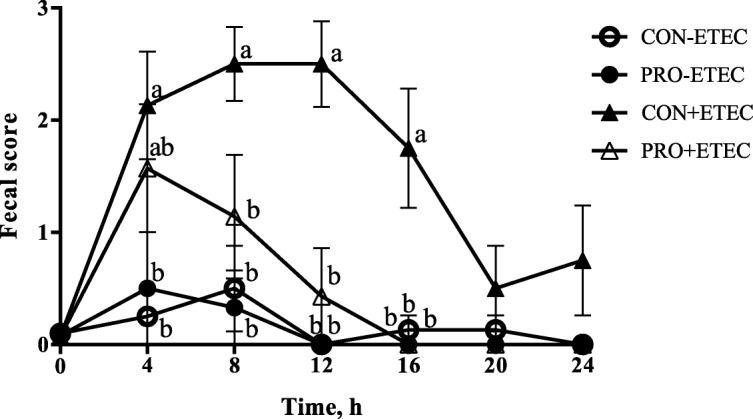
Effect of *E. faecium* on fecal score of piglets challenged with ETEC K88. Values are means, with their standard errors represented by vertical bars (*n* = 8). CON-ETEC, control group infusing the essential medium; CON+ETEC, control group infusing the *Escherichia coli*; PRO-ETEC, *Enterococcus faecium*-supplemented group infusing the essential medium; PRO+ETEC, *Enterococcus faecium*-supplemented group infusing the *Escherichia coli*. Fecal score: 0, normal; 1, pasty; 2, semiliquid; and 3, liquid. Time, hours after ETEC challenge. ^a, b ^Means in the same time with different superscripts are significantly different (*P* < 0.05)

As shown in Fig. 1, piglets challenged with ETEC had greater fecal score than that of non-challenged piglets at 4, 8, 12, and 16 h post challenge (*P* <  0.05), whereas the PRO administration was able to alleviate the fecal score at 12 and 16 h post challenge (*P* < 0.05). In addition, there was an interaction between PRO and ETEC challenge at 12 h and 16 h post challenge (*P* < 0.05).

### Routine blood examination and composition of peripheral lymphocyte percentages

**Fig. 2:**
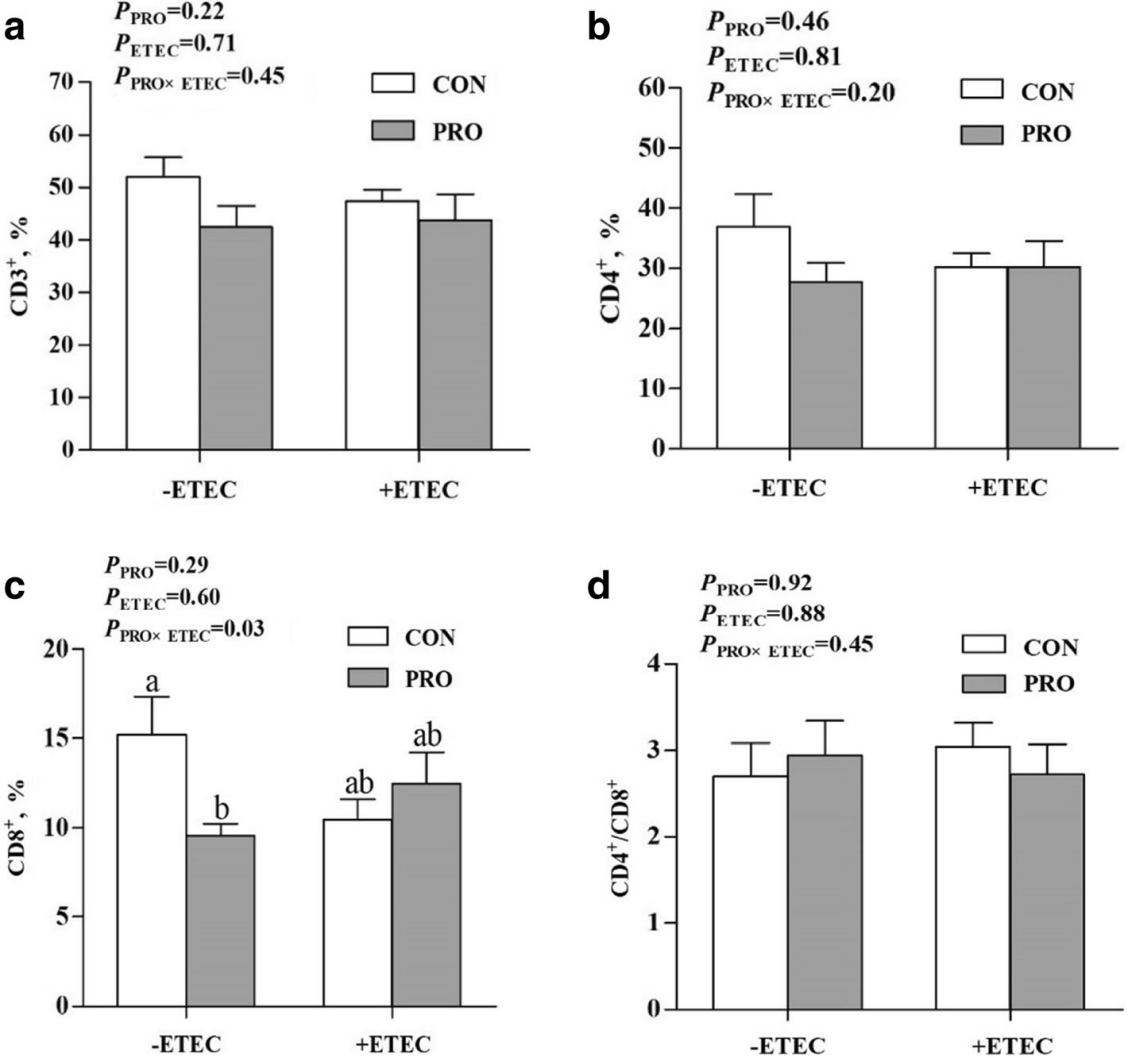
Effect of *E. faecium* on the composition of peripheral lymphocyte percentages of piglets challenged with ETEC K88. **a**, percentage of CD3^+^ T-lymphocytes; **b**, percentage of CD4^+^ T-lymphocytes; **c**, percentage of CD8^+^ T-lymphocytes; **d**, ratio of CD4^+^ to CD8^+^. -ETEC, infusing the essential medium; +ETEC, infusing the *Escherichia coli*; CON, control group; PRO, *Enterococcus faecium*-supplemented group. Values are means, with their standard errors represented by vertical bars (*n* = 8). ^a, b^ Mean values with different superscript letters are significantly different (*P* < 0.05)

ETEC challenge markedly increased the count and percentage of lymphocytes (*P* < 0.05) (Table 3). The count and percentage of lymphocytes were increased (*P* < 0.05) in the CON+ETEC group than in the CON-ETEC group, whereas no significant difference was observed between the PRO groups. The composition of peripheral lymphocyte percentages was not markedly affected by PRO (*P* > 0.10) or ETEC (*P* > 0.10), whereas there was an interaction between PRO and ETEC challenge for the percentage of CD8^+^ T cells (*P* < 0.05) (Fig. 2). The percentage of CD8^+^ T cells was lower (*P* < 0.05) in the PRO-ETEC group than that in the CON-ETEC group.

**Table 3:** Effect of *E. faecium* on blood routine parameters of piglets challenged with ETEC K88

Items	-ETEC		+ETEC		*P*-value		
	CON	PRO	CON	PRO	PRO	ETEC	PRO×ETEC
White blood cell,10^9^/L	7.26 ± 1.20	8.80 ± 1.67	7.77 ± 0.98	8.16 ± 1.22	0.45	0.96	0.65
Lymphocytes,10^9^/L	1.46 ± 0.16^b^	1.85 ± 0.19^b^	2.96 ± 0.62^a^	2.60 ± 0.51^ab^	0.97	0.03	0.44
Neutrophils,10^9^/L	4.55 ± 0.62	4.76 ± 0.49	4.23 ± 0.77	5.23 ± 0.49	0.36	0.91	0.54
Intermediate cell, 10^9^/L	0.40 ± 0.04	0.65 ± 0.20	0.59 ± 0.13	0.56 ± 0.14	0.44	0.74	0.35
Lymphocytes, %	21.45 ± 2.51^b^	25.05 ± 3.58^ab^	37.24 ± 5.83^a^	29.23 ± 2.60^ab^	0.61	0.03	0.19
Neutrophils, %	69.40 ± 2.42	58.28 ± 6.87	54.73 ± 6.71	63.42 ± 3.75	0.82	0.39	0.08
Intermediate cell, %	6.04 ± 1.16	6.77 ± 1.31	8.03 ± 1.71	6.12 ± 0.87	0.70	0.66	0.38
Red blood cell, 10^12^/L	9.17 ± 0.37	8.77 ± 0.67	9.35 ± 0.47	9.20 ± 0.31	0.57	0.52	0.80
Haematocrit, %	78.34 ± 3.00	73.35 ± 6.70	76.69 ± 2.46	76.30 ± 1.22	0.44	0.85	0.51
Mean corpuscular volume, fL	85.56 ± 0.70	83.60 ± 3.81	82.61 ± 2.12	83.28 ± 2.21	0.79	0.50	0.58
Platelet, 10^9^/L	576.00 ± 134.95	441.50 ± 82.92	508.42 ± 109.58	636.20 ± 123.37	0.98	0.61	0.29
Thrombocytocrit, %	0.72 ± 0.24	0.45 ± 0.10	0.61 ± 0.17	0.79 ± 0.23	0.82	0.60	0.29

Data are presented as means ± SE (*n* = 8)

-ETEC, infusing the essential medium; +ETEC, infusing the *Escherichia coli*; CON, control group; PRO, *Enterococcus faecium*-supplemented group

^a, b^Means within a row with different superscripts are significantly different (*P* < 0.05)

### Inflammatory cytokine concentrations

As shown in Table 4, ETEC challenge elevated the concentrations of IL-1β in plasma and TNF-α in ileal mucosa (*P* < 0.05). The concentrations of IL-1β in plasma and TNF-α in ileal mucosa were increased (*P* < 0.05) in the CON+ETEC group than that in the CON-ETEC group, whereas no significant difference was observed between the PRO+ETEC and PRO-ETEC groups.

**Table 4:** Effect of *E. faecium* on cytokines concentrations in the plasma and ileal mucosa of piglets challenged with ETEC K88

Items	-ETEC		+ETEC		*P*-value		
	CON	PRO	CON	PRO	PRO	ETEC	PRO×ETEC
Plasma							
IL-1β, ng/mL	0.78 ± 0.07^b^	0.93 ± 0.15^ab^	1.32 ± 0.20^a^	1.13 ± 0.21^ab^	0.93	0.04	0.33
IL-6, ng/mL	11.04 ± 1.25	11.07 ± 1.90	11.31 ± 1.03	12.32 ± 1.47	0.71	0.59	0.72
TNF-α, pg/mL	49.16 ± 6.44	51.29 ± 5.42	57.20 ± 6.50	42.81 ± 4.53	0.38	0.97	0.24
Ileal mucosa							
IL-1β, ng/mL	5.22 ± 0.85	5.08 ± 0.51	5.67 ± 0.92	5.87 ± 0.75	0.97	0.45	0.83
IL-6, ng/mL	9.22 ± 0.74	10.30 ± 0.42	11.04 ± 0.59	10.84 ± 0.79	0.54	0.11	0.37
TNF-α, ng/mL	0.90 ± 0.06^b^	0.88 ± 0.06^b^	1.15 ± 0.07^a^	1.02 ± 0.06^ab^	0.25	< 0.01	0.41

Data are presented as means ± SE (*n* = 8)

-ETEC, infusing the essential medium; +ETEC, infusing the *Escherichia coli*; CON, control group; PRO, *Enterococcus faecium*-supplemented group

^a, b^Means within a row with different superscripts are significantly different (*P* < 0.05)

### Intestinal morphology and goblet cell density

**Fig. 3:**
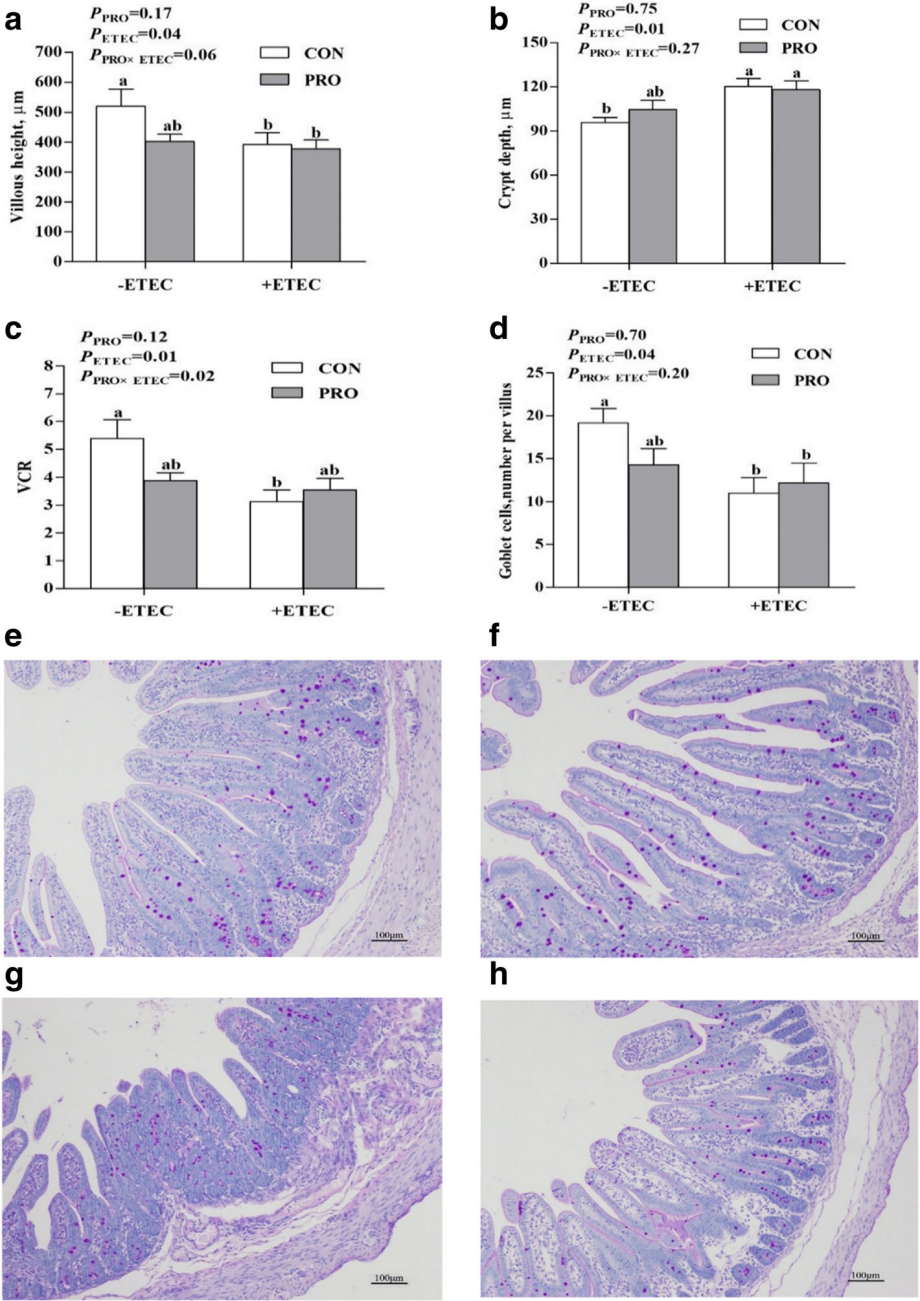
Effect of *E. faecium* on the intestinal histomorphology and number of goblet cells in the jejunum of piglets challenged with *Escherichia coli* (ETEC) K88. **a**, villous height; **b**, crypt depth; **c**, the ratio of villous height:crypt depth (VCR); **d**, number of goblet cells; **e-h**, Representative micrographs of goblet cell staining carried out on paraformaldehyde-fixed sections from the jejunum (100× magnification) of piglets challenged with ETEC K88 (**e**, CON-ETEC; **f**, PRO-ETEC; **g**, CON+ETEC; **h**, PRO+ETEC). -ETEC, infusing the essential medium; +ETEC, infusing the *Escherichia coli*; CON, control group; PRO, *Enterococcus faecium*-supplemented group; VCR, Villous height:crypt depth ratio. Values are means, with their standard errors represented by vertical bars (*n* = 8). ^a,b^Means values with different superscript letters are significantly different (*P* < 0.05). For VCR (**c**), the superscript letters referred to significant effect of ETEC

The ETEC challenge reduced villous height and the ratio of villous height:crypt depth (VCR) of jejunum (*P* < 0.05) and increased crypt depth of jejunum (*P* < 0.05) (Fig. 3a-c). There was an interaction between PRO and ETEC challenge for jejunal VCR (*P* < 0.05). Jejunal VCR was lower (*P* < 0.05) in the CON+ETEC group than in the CON-ETEC group, whereas jejunal VCR did not differ (*P* > 0.05) between the PRO+ETEC and PRO-ETEC groups. The number of goblet cells in the jejunum was decreased (*P* < 0.05) by ETEC challenge (Fig. 3d-h). Following ETEC challenge, jejunal goblet cell numbers were decreased (*P* < 0.05) in the CON groups, whereas no significant difference was observed between the PRO groups.

### Gene expression in the ileum

**Fig. 4:**
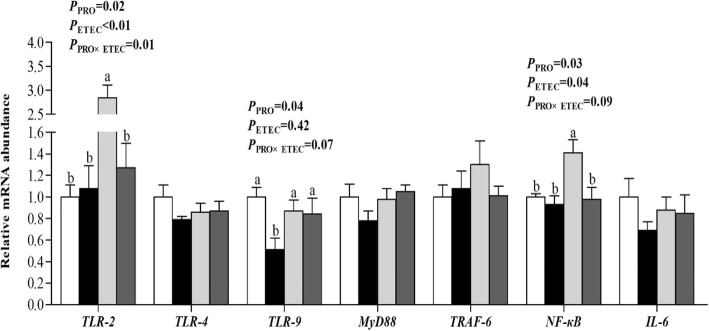
Effect of *E. faecium* on mRNA abundance of innate immune-related genes in the ileum of piglets challenged with ETEC K88. Values are means, with their standard errors represented by vertical bars (*n* = 8). , CON-ETEC; ,PRO-ETEC; , CON+ETEC; , PRO+ETEC. *TLR*, Toll-like receptor; *MyD88*, myeloid differentiation factor 88; *TRAF-6*, TNF receptor-associated factor 6; *NF-κB*, nuclear transcription factor kappa B; *IL*, interleukin. ^a, b^ Mean values with different superscript letters are significantly different (*P* < 0.05). For *TLR-2* and *NF-κB*, the superscript letters referred to significant effect of ETEC

**Fig. 5:**
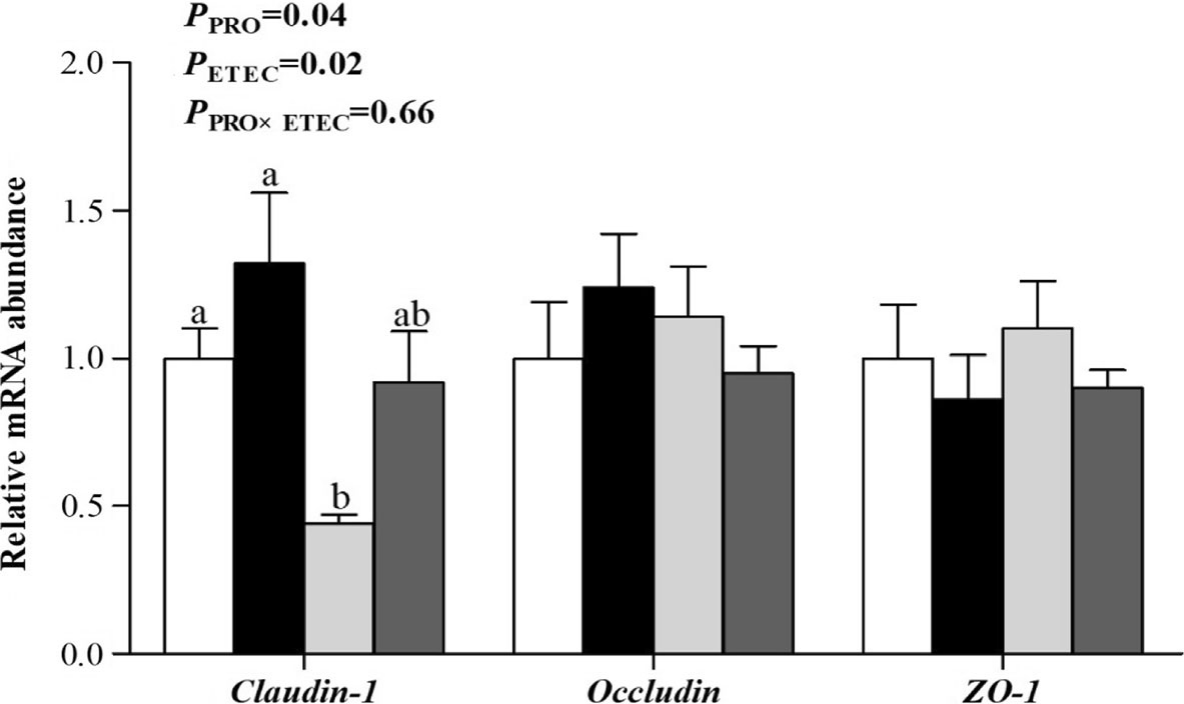
Effect of *E. faecium* on mRNA abundance of tight junction proteins in the ileum of piglets challenged with ETEC K88. Values are means, with their standard errors represented by vertical bars (*n* = 8)., CON-ETEC;,PRO-ETEC;, CON+ETEC;, PRO+ETEC. *ZO-1*, zonula occludens-1. ^a, b^ Means values with different superscript letters are significantly different (*P* < 0.05). For *claudin-1*, the superscript letters referred to significant effect of ETEC

The mRNA abundances of *TLR-2* and *NF-κB* (*P* < 0.05) in the ileum were increased (*P* < 0.05) by ETEC challenge (Fig. 4). Following ETEC challenge, the mRNA abundances of *TLR-2* and *NF-κB* were increased (*P* < 0.05) in the CON groups, whereas no difference was observed between the PRO groups. Ileal *TLR-2* mRNA abundance was affected by PRO (*P* < 0.05) and the PRO × ETEC interaction (*P* < 0.05). In addition, piglets in PRO groups had lower mRNA abundances of *TLR-9* and *NF-κB* (*P* < 0.05) in the ileum when compared to those in CON groups. Furthermore, piglets challenged with ETEC had lower mRNA abundance of *claudin-1* (*P* < 0.05) in the ileum, and the PRO supplementation was able to increase the mRNA abundance of *claudin-1* (*P* < 0.05, Fig. 5). The mRNA abundance of *Claudin-1* was decreased (*P* < 0.05) in the CON+ETEC group than in the CON-ETEC group, whereas no difference was observed between the PRO groups.

### Gut bacterial community structure

A total of 1,943,803 high-quality sequences were obtained from 4 groups, with an average of 80,991 sequences per sample. All of OTUs were defined at 97% species similarity level, 12,894 OTUs were obtained from colonic digesta samples, with an average of 537 OTUs per sample. Four alpha diversity measures were calculated including observed species, Shannon index, Chao 1 index, and phylogenetic diversity (PD) tree (Additional file 1: Table S1). However, these measures were not significantly affected by PRO, ETEC challenge, or the interaction between PRO and ETEC challenge. In addition, the relationships among bacterial communities from different treatments were represented by principal coordinate analysis (PCoA), and the results showed that the microbial communities of piglets in the four groups were not significantly different (Additional file 1: Figure S2).

At the phylum level, there were six phyla with a relative abundance greater than 0.5% in at least one experimental group: Proteobacteria, Bacteroidetes, Firmicutes, Fusobacteria, Planctomycetes, Lentisphaerae (Table 5). Of these six phyla, Bacteroidetes predominated in all samples, with a relative abundance of 42.27%–47.63%, followed by Firmicutes, at 19.03%–33.46%. Piglets challenged with ETEC tended to increase (*P* = 0.07) the relative abundance of Proteobacteria, and the PRO supplementation increased (*P* = 0.05) the relative abundance of Verrucomicrobia.

**Table 5:** Effect of *E. faecium* on the relative abundance of the top 10 microbial phylum in the colon of piglets challenged with ETEC K88

Items	-ETEC		+ETEC		*P*-value		
	CON	PRO	CON	PRO	PRO	ETEC	PRO×ETEC
Proteobacteria	15.91 ± 2.91	12.06 ± 2.40	18.38 ± 1.41	17.81 ± 2.91	0.23	0.07	0.48
Bacteroidetes	47.63 ± 3.45	44.04 ± 3.53	47.42 ± 4.38	42.27 ± 5.95	0.29	0.68	0.75
Firmicutes	24.40 ± 4.19	33.46 ± 8.34	19.03 ± 1.80	29.66 ± 7.07	0.37	0.52	0.95
Fusobacteria	11.25 ± 4.60	9.06 ± 5.56	14.73 ± 5.02	9.29 ± 6.97	0.11	0.98	0.67
Verrucomicrobia	0.01 ± 0.01	0.01 ± 0.00	0.02 ± 0.02	0.25 ± 0.23	0.05	0.82	0.53
Planctomycetes	0.04 ± 0.02	0.69 ± 0.64	0.06 ± 0.04	0.11 ± 0.08	0.24	0.14	0.68
Lentisphaerae	0.51 ± 0.30	0.29 ± 0.09	0.13 ± 0.11	0.25 ± 0.13	0.23	0.11	0.43
Spirochaetes	0.03 ± 0.02	0.14 ± 0.10	0.03 ± 0.02	0.12 ± 0.06	0.92	0.59	0.64
Euryarchaeota	0.08 ± 0.04	0.07 ± 0.05	0.07 ± 0.06	0.10 ± 0.05	0.42	0.98	0.58
Actinobacteria	0.07 ± 0.03	0.09 ± 0.04	0.04 ± 0.01	0.08 ± 0.03	0.29	0.47	0.59

Data are presented as means ± SE (*n* = 6)

-ETEC, infusing the essential medium; +ETEC, infusing the *Escherichia coli*; CON, control group; PRO, *Enterococcus faecium*-supplemented group

**Fig. 6:**
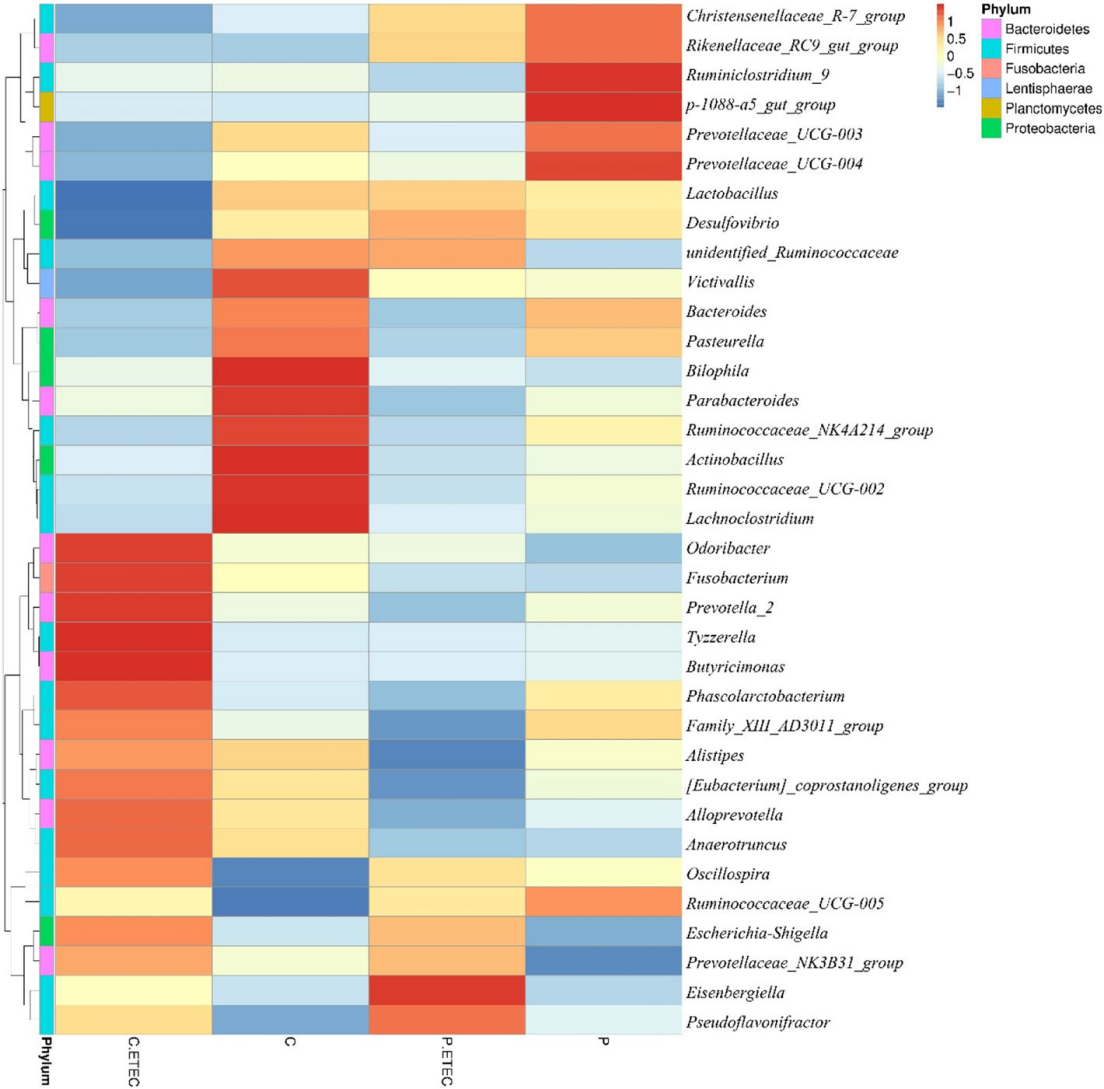
Effect of *E. faecium* on the relative abundance of microbial genera in the colon of piglets challenged with ETEC K88. The relative percentages (%) of the bacterial genus are indicated by varying color intensities according to the legend at the top of the figure. Bacterial genus names are listed on the right side of the heatmap, and the phylum names are listed on the left. The intensity of the cell color represents the abundance of the amplicons belonging to each genus. C.ETEC, CON+ETEC; C, CON-ETEC; P.ETEC, PRO+ETEC; P, PRO-ETEC

The heatmap in Fig. 6 shows the relative abundances of various bacteria at the genus level in the different groups. Compared with CON groups, piglets in PRO groups had lower abundance of *Bilophila* (*P* < 0.05), and had a tendency for lower abundance of *Parabacteroides* (*P* = 0.06). In addition, piglets challenged with ETEC had lower abundance of *Lachnoclostridium* (*P* < 0.05), and tended to decrease the abundance of *Ruminococcaceae_NK4A214_group* (*P* = 0.07), *Prevotella_7* (*P* = 0.08) and *Lactobacillus* (*P* = 0.08), whereas the relative abundance of *Escherichia-Shigella* and *Prevotellaceae_NK3B31_group* were significantly increased (*P* ≤ 0.05). Meanwhile, the decreased *Lachnoclostridium* at genus level caused by ETEC were relieved by feeding *E. faecium* (Additional file 1: Table S2).

### SCFAs concentrations

As shown in Additional file 1: Table S3, concentrations of acetate, propionic acid and butyric acid in colonic digesta were not markedly affected by PRO or ETEC challenge (*P* > 0.05).

## Discussion

Oral administration of *E. faecium* probiotics in humans has been considered to be effective in the prevention of antibiotic-associated diarrhea [36] and in the treatment of diarrhoeal disorders in children [37]. In addition, *E. faecium* has been used as a probiotic in livestock animal to improve growth performance and intestinal health [38], and reduce diarrhea incidence [17, 19]. Büsing and Zeyner suggested that oral administration of *E. faecium* NCIMB 10415 could improve the growth rate and mitigate incidence and severity of diarrhoea in suckling piglets [13]. However, some researchers suggested *E. faecium* failed to affect the growth performance of piglets [39, 40]. In the current study, we found the oral administration of *E. faecium* did not show beneficial effects on the growth rate and diarrhea severity of 2- to 8-day-old piglets. The discrepancies between studies might be due to different strains and dose of *E. faecium*, as well as the length of time *E. faecium* was supplied. It is considered that growth promoters are more effective when the livestock animals suffered environmental and nutritional challenges [39]. Accordingly, the positive effect of *E. faecium* may be obvious under the condition of pathogenic pressure, which was confirmed in the ETEC challenge study, indicating that oral administration of *E. faecium* attenuated the diarrhea severity in sucking piglets challenged by ETEC.

The villous height and the crypt depth are important indicators to reflect the digestive and absorptive functions of the small intestine [41]. The shortening of the villous height may imply the decreased surface area for nutrient absorption, and a deeper crypt may suggest a faster turnover of new villous cells [42]. The VCR is a useful indicator for assessing intestinal function and health [43]. This study showed that piglets challenged with ETEC had deeper crypts, and reduced VCR in the jejunum. However, the oral administration of *E. faecium* could attenuate the effect of ETEC challenge on the morphology of jejunum, which is in accordance with the lower diarrhea score in piglets supplemented with *E. faecium* during the 24-h post challenge. Supportively, Xie et al. reported that piglets fed the diet containing *E. faecium* had increased villus height in the jejunum and reduced crypt depth in the ileum [38].

Infection with ETEC is often associated with diarrhea and impaired intestinal barrier function. Goblet cells producing mucins are the important component of non-specific intestinal barrier, partly protecting animals against bacterial and fungal invasion [44]. The decrease in number of goblet cells may decrease the mucin secretion of mucosa [45], which would be detrimental for the mucosal barrier. In this study, dietary *E. faecium* supplementation attenuated the effect of ETEC challenge on the number of goblet cells, which suggests the effect of *E. faecium* supplementation on improving mucosal barrier of piglets under ETEC challenge. Likewise, the oral administration of *E. faecium* increased mRNA abundance of *claudin-1* in the ileum of sucking piglets. Claudin-1, ZO-1 and occludin are the most important components in the structural and functional organization of epithelial tight junctions [46]. Hence, the increased expression of *claudin-1* suggests the better intestinal barrier function in response to dietary supplementation of *E. faecium*. Supportively, data from IPEC-J2 cell line indicated that *E. faecium* was able to increase the transepithelial electrical resistance of enterocyte monolayer, thus strengthening the intestinal barrier against ETEC [47].

Immunologically, the increased plasma IL-1β concentration and blood lymphocyte count indicated the successful establishment of immune model following ETEC challenge. Immunocyte number and variation reflect the immunity or infection status, an increase in the count and percentage of lymphocytes indicate inflammation [48, 49]. The increased percentage of lymphocytes in the blood has been shown in piglets with ETEC challenge [49]. In this study, however, we found oral administration of *E. faecium* did not markedly alter the percentage of lymphocytes in the ETEC-challenged piglets, indicating *E. faecium* may have an anti-inflammatory effect against ETEC. Similarly, Tian et al. reported that *E. faecium* could effectively inhibit intestinal inflammation caused by ETEC [47]. T lymphocytes are responsible for cell-mediated immunity, and it can be divided into subsets according to the presence of CD4 and CD8 proteins [50]. The main function of CD4^+^ T cells is to direct the immune response towards invading pathogens and tumorigenic cells, and to maintain immune homeostasis [51], while CD8^+^ T cells play a pivotal role in the control of viral infections and tumor cells, for antigen-specific responses against multifarious pathogens and vaccine-induced immunity [52]. Previous study has shown that early administration of the probiotic *E. faecium* can modulate the composition of blood lymphocyte populations in sucking piglets [53]. Our study demonstrated that piglets in the PRO-ETEC group had lower percentage of CD8^+^ T cells compared with piglets in the CON-ETEC group. Similarly, there was a suppressive effect of the probiotic *E. faecium* on the CD8^+^ T cells, associating with a remarkable decrease in the colonization of pathogenic bacteria [16, 54].

The intestine is the largest immunological organ in the body, and as such is the location for the majority of lymphocytes and immune effector cells with pattern-recognition receptors [55]. Toll-like receptors (TLRs) are typical pattern recognition receptors in mediating mucosal innate host defense to maintain mucosal and commensal homeostasis [56]. It is well documented that the diarrhea and impaired intestinal barrier integrity are often associated with the activation of innate immunity and inflammatory response, in which the TLR/MyD88/NF-κB signal pathway are involved [57, 58]. It has been reported that ETEC expressing K88 fimbriae mediated bacterial adherence to host cells, which would activate innate immune response by delivering microbial associated molecular pattern products, such as LPS or fimbriae-dependent signaling K88, involving TLRs as pattern recognition receptors [59]. In the present study, our data indicated that ETEC infection stimulated the mRNA abundances of *TLR2* and *NF-κB* in the ileum of ETEC-challenged piglets, which were significantly down-regulated by the oral administration of *E. faecium*. Similarly, a previous study also showed that dietary supplementation of *Lactobacillus acidophilus* could alleviate the inflammatory response by inhibiting ETEC-induced TLR2 expression, associating with the down-regulated NF-κB and MAPK signaling pathways in piglets [60]. Besides, the activated TLRs would stimulate the NF-ĸB signal pathway and then increase the expression of various inflammatory cytokines, including IL-1β, TNF-α and IL-6, etc. [60, 61]. In the present study, we found that ETEC challenge increased the level of TNF-α in the ileal mucosa. Importantly, piglets in the PRO+ETEC group normalized the concentration of TNF-α to be similar to those piglets in the PRO-ETEC group. Considering the crucial role of cytokines in immune and inflammatory responses [62], these findings indicate that oral administration of *E. faecium* may have beneficial effects in reducing intestinal inflammation in ETEC-challenged piglets.

The relationship between the gut microbiota and animal health is being extensively investigated. Intestinal microorganisms play critical roles in the development of host immune system [63]. During the early neonatal period, gut microbiota is unstable and prone to be modified [64, 65]. Therefore, *E. faecium* administration to neonatal piglets may modify the composition of the gut microbiota for intestinal health. In this study, *E. faecium* administration and ETEC challenge did not affect the microbiota richness and diversity of suckling piglets. At the phylum level, however, the abundance of Proteobacteria tended to increase in piglets with ETEC challenge, and *E. faecium* supplementation significantly increased the abundance of Verrucomicrobia. Proteobacteria contains many enteric pathogens, such as *Salmonella* and *Escherichia*, which might cause diarrhea [19]. An increased prevalence of the phylum Proteobacteria in the gut reflects dysbiosis or unstable gut microbial community structure [66]. At the genus level, ETEC infection increased the abundance of *Escherichia-Shigella* in ETEC-challenged piglets. In contrast, a high abundance of Verrucomicrobia has been proposed as a hallmark of healthy gut due to its benefits on anti-inflammation and intestinal barrier function [67]. In this study, the increased abundance of Verrucomicrobia by *E. faecium* favor the colonic barrier function.

At the genus level, furthermore, piglets in the PRO-ETEC group had lower abundance of *Bilophila* compared with piglets in the CON-ETEC group. A previous study has indicated that *Bilophila* is detected as a high abundant microbe in pathological conditions such as colitis and other intestinal inflammatory disorders [68]. The potential mechanism *Bilophila* causing intestinal inflammation is the production of sulfide that breaks the mucus barrier, thereby allowing close proximity of bacteria to the epithelium with epithelial damage and inflammation [69]. In our study, moreover, we found that piglets in the PRO+ETEC group normalized the relative abundance of *Lachnoclostridium* to be similar to those piglets in the PRO-ETEC group. Although *Lachnoclostridium* is responsible for SCFAs production [70], no significant differences were observed for the SCFAs contents in the digesta of the colon among groups. In addition, oral administration of *E. faecium* tended to decrease the abundance of *Parabacteroides*, which was taken as opportunistic pathogens in infectious diseases, and are able to develop antimicrobial drug resistance [71]. *Parabacteroides* spp. had a negative correlation with colonic expression of tight junction protein and anti-inflammatory protein genes [72]. This was consistent with our study that *E. faecium* administration improved the intestinal barrier function and immune function. The collective data described above suggest that oral administration of *E. faecium* could manipulate the microbiota profile with a decrease in pathogenic bacteria and an increase in beneficial bacteria, supporting the phenotype of intestinal health.

## Conclusion

Our results indicate that oral administration of *E. faecium* to neonatal piglets had no significant effect on growth performance, but relieved the negative effects of ETEC infection on diarrhea, intestinal morphology and immunology of piglets, which could be partly ascribed to the changes in the intestinal microbiota profile.

## Additional file

Additional file 1: **Table S1.** Effect of *E. faecium* on alpha diversity of microbial community in colonic content of piglets challenged with ETEC K88. **Table S2.** Effect of *E. faecium* on the relative abundance for the top 30 most abundant genera in the colon of piglets challenged with ETEC K88. **Table S3.** Effect of *E. faecium* on short chain fatty acid concentrations of piglets challenged with ETEC K88. **Figure S1.** The experimental design of the different treatments and procedures. **Figure S2.** Comparison of the gut microbiota composition among four groups. Principal coordinate analysis to visualize the unweighted UniFrac distances of colon digesta samples from individual piglet. (DOCX 137 kb)

## Data Availability

The datasets analyzed in the current study are available from the corresponding author on reasonable request.

## Abbreviations

ADG: Average daily gain
BW: Body weight
CON: Control group
ETEC: Enterotoxigenic *Escherichia coli*
IL: Interleukin
MyD88: Myeloid differentiation factor 88
NF-κB: Nuclear transcription factor kappa B
PRO: *Enterococcus faecium*-supplemented group
TLR: Toll-like receptor
TRAF-6: TNF receptor-associated factor 6
VCR: The ratio of villous height:crypt depth
ZO-1: Zonula occludens-1
