# Supplementary material for: Enterococcus faecium NCIMB 10415 administration improves the intestinal health and immunity in neonatal piglets infected by enterotoxigenic Escherichia coli K88
Source: J Anim Sci Biotechnol. 2019 Aug 21;10:72. doi: 10.1186/s40104-019-0376-z (PMC6702752; doi:10.1186/s40104-019-0376-z)

*Supplementary Material*

**Table S1.** Effect of *E. faecium* on alpha diversity of microbial community in colonic content of piglets challenged with ETEC K88

|  | -ETEC | |  | +ETEC | |  | *P*-value | | |
| --- | --- | --- | --- | --- | --- | --- | --- | --- | --- |
|  | CON | PRO |  | CON | PRO |  | PRO | ETEC | PRO×ETEC |
| Observed-species | 505.17±24.98 | 502.50±21.53 |  | 482.83±25.60 | 493.83±24.93 |  | 0.87 | 0.53 | 0.78 |
| Shannon | 5.97±0.16 | 5.73±0.24 |  | 5.90±0.21 | 5.59±0.18 |  | 0.19 | 0.62 | 0.86 |
| Chao1 | 541.10±27.70 | 530.41±19.46 |  | 514.85±25.45 | 536.56±23.34 |  | 0.82 | 0.68 | 0.51 |
| PD whole tree | 31.40±1.58 | 31.42±1.09 |  | 29.58±1.43 | 30.28±1.37 |  | 0.79 | 0.29 | 0.81 |

Data are presented as means ± SE (*n* = 6).

-ETEC, infusing the essential medium; +ETEC, infusing the *Escherichia coli*; CON, control group; PRO, *Enterococcus faecium*-supplemented group.

**Table S2.** Effect of *E. faecium* on the relative abundance for the top 30 most abundant genera in the colon of piglets challenged with ETEC K88

|  | -ETEC | | |  | +ETEC | |  | *P*-value | | |
| --- | --- | --- | --- | --- | --- | --- | --- | --- | --- | --- |
|  | CON | PRO |  | | CON | PRO |  | PRO | ETEC | PRO×ETEC |
| *Fusobacterium* | 10.91±4.57 | 8.81±5.36 |  | | 14.68±5.04 | 8.96±6.95 |  | 0.11 | 0.97 | 0.64 |
| *Escherichia-Shigella* | 9.99±3.86^ab^ | 8.38±2.03^b^ |  | | 15.38±1.26^a^ | 14.45±3.10^ab^ |  | 0.69 | 0.02 | 0.84 |
| *Prevotella_2* | 7.38±2.13 | 7.74±2.92 |  | | 14.06±3.46 | 4.88±1.07 |  | 0.32 | 0.29 | 0.42 |
| *Bacteroides* | 8.14±1.97 | 7.61±3.12 |  | | 5.19±1.19 | 5.14±2.01 |  | 0.40 | 0.34 | 0.80 |
| *Alloprevotella* | 5.99±1.67 | 4.88±1.04 |  | | 7.08±1.99 | 4.04±0.96 |  | 0.32 | 0.82 | 0.48 |
| *Butyricimonas* | 1.66±0.44 | 1.81±0.52 |  | | 4.44±1.32 | 1.68±0.41 |  | 0.18 | 0.14 | 0.10 |
| *Rikenellaceae_RC9_gut_group* | 3.49±0.85 | 4.41±0.77 |  | | 3.49±0.74 | 4.14±1.42 |  | 0.46 | 0.70 | 0.58 |
| *Lachnoclostridium* | 4.86±1.23^a^ | 2.63±0.46^ab^ |  | | 1.99±0.45^b^ | 2.23±0.68^b^ |  | 0.26 | 0.02 | 0.28 |
| *Prevotellaceae_UCG-003* | 1.24±1.11 | 1.49±1.25 |  | | 0.57±0.24 | 0.80±0.42 |  | 0.87 | 0.56 | 0.89 |
| *Ruminococcaceae_UCG-002* | 1.90±0.95 | 1.55±0.66 |  | | 1.44±0.52 | 1.44±0.65 |  | 0.95 | 0.83 | 0.92 |
| *Prevotellaceae_NK3B31_group* | 1.30±0.88^b^ | 0.74±0.16^ab^ |  | | 1.76±0.23^a^ | 1.70±0.79^ab^ |  | 0.95 | 0.05 | 0.32 |
| *Actinobacillus* | 2.49±0.96 | 1.17±0.21 |  | | 0.97±0.39 | 0.86±0.37 |  | 0.89 | 0.29 | 0.63 |
| *Tyzzerella* | 0.14±0.07 | 0.22±0.10 |  | | 1.50±0.90 | 0.15±0.08 |  | 0.57 | 0.57 | 0.31 |
| *Parabacteroides* | 3.13±0.45 | 1.91±0.61 |  | | 1.86±0.42 | 1.39±0.41 |  | 0.06 | 0.10 | 0.63 |
| *Alistipes* | 1.47±0.62 | 1.23±0.49 |  | | 1.60±0.51 | 0.76±0.21 |  | 0.17 | 0.68 | 0.32 |
| *Eubacterium_coprostanoligenes_group* | 0.91±0.54 | 0.77±0.47 |  | | 1.08±0.66 | 0.52±0.23 |  | 0.91 | 0.95 | 0.77 |
| *p-1088-a5_gut_group* | 0.04±0.02 | 0.69±0.64 |  | | 0.06±0.04 | 0.12±0.07 |  | 0.26 | 0.12 | 0.70 |
| *Ruminococcaceae_NK4A214_group* | 1.75±0.53 | 1.35±0.13 |  | | 1.04±0.35 | 1.05±0.36 |  | 0.89 | 0.07 | 0.97 |
| *Pasteurella* | 0.73±0.49 | 0.59±0.46 |  | | 0.18±0.07 | 0.20±0.10 |  | 0.99 | 0.35 | 0.85 |
| *Lactobacillus* | 1.44±0.33 | 1.30±0.35 |  | | 0.44±0.22 | 1.44±0.44 |  | 0.16 | 0.08 | 0.11 |
| *Anaerotruncus* | 0.40±0.13 | 0.14±0.03 |  | | 0.55±0.42 | 0.12±0.03 |  | 0.15 | 0.45 | 0.64 |
| *Oscillospira* | 0.69±0.17 | 0.73±0.15 |  | | 0.76±0.13 | 0.75±0.36 |  | 0.52 | 0.73 | 0.39 |
| *unidentified_Ruminococcaceae* | 0.78±0.32 | 0.45±0.14 |  | | 0.40±0.14 | 0.76±0.36 |  | 0.92 | 0.66 | 0.23 |
| *Bilophila* | 0.87±0.33^a^ | 0.17±0.05^b^ |  | | 0.27±0.09^ab^ | 0.24±0.08^b^ |  | 0.04 | 0.29 | 0.22 |
| *Desulfovibrio* | 0.89±0.28 | 0.90±0.21 |  | | 0.74±0.16 | 0.93±0.13 |  | 0.37 | 0.93 | 0.57 |
| *Eisenbergiella* | 0.23±0.05 | 0.21±0.09 |  | | 0.34±0.13 | 0.58±0.33 |  | 0.80 | 0.44 | 0.49 |
| *Odoribacter* | 0.26±0.12 | 0.09±0.05 |  | | 0.62±0.33 | 0.24±0.07 |  | 0.28 | 0.15 | 0.41 |
| *Ruminococcaceae_UCG-005* | 0.21±0.07 | 0.47±0.30 |  | | 0.38±0.18 | 0.40±0.19 |  | 0.74 | 0.87 | 0.86 |
| *Victivallis* | 0.44±0.30 | 0.20±0.09 |  | | 0.03±0.01 | 0.22±0.13 |  | 0.19 | 0.11 | 0.25 |
| *Prevotella_7* | 0.04±0.03 | 0.37±0.28 |  | | 0.00±0.00 | 0.03±0.03 |  | 0.18 | 0.08 | 0.35 |

Data are presented as means ± SE (*n* = 6).

-ETEC, infusing the essential medium; +ETEC, infusing the *Escherichia coli*; CON, control group; PRO, *Enterococcus faecium*-supplemented group.

^a, b^ Means within a row with different superscripts are significantly different (*P* < 0.05)

**Table S3.** Effect of *E. faecium* on short chain fatty acid concentrations of piglets challenged with ETEC K88

|  | -ETEC | |  | +ETEC | |  | *P*-value | | |
| --- | --- | --- | --- | --- | --- | --- | --- | --- | --- |
|  | CON | PRO |  | CON | PRO |  | PRO | ETEC | PRO×ETEC |
| Acetate, mg/g | 0.61±0.07 | 0.59±0.05 |  | 0.73±0.09 | 0.61±0.12 |  | 0.39 | 0.39 | 0.58 |
| Propionic acid, mg/g | 0.25±0.03 | 0.23±0.04 |  | 0.33±0.05 | 0.21±0.04 |  | 0.15 | 0.27 | 0.32 |
| Butyric acid, mg/g | 0.11±0.02 | 0.09±0.02 |  | 0.14±0.02 | 0.09±0.03 |  | 0.20 | 0.27 | 0.71 |

Data are presented as means ± SE (*n* = 8).

-ETEC, infusing the essential medium; +ETEC, infusing the *Escherichia coli*; CON, control group; PRO, *Enterococcus faecium*-supplemented group.

**Figure S1.** The experimental design of the different treatments and procedures.


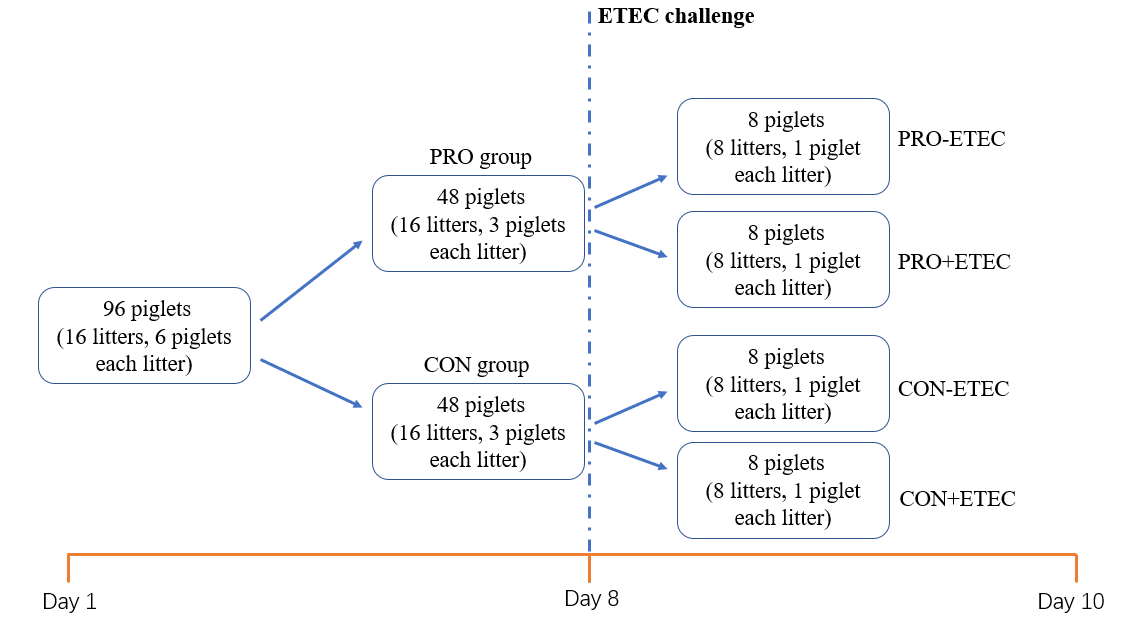


Day 1-7: treatment with *E. faecium* for one week

Day 8: ETEC challenge only at day 8

Day 10: tissue sample collection

**Figure S2.** Comparison of the gut microbiota composition among four groups. Principal coordinate analysis to visualize the unweighted UniFrac distances of colon digesta samples from individual piglet.

C.ETEC: CON+ETEC; C: CON-ETEC; P.ETEC: PRO+ETEC; P: PRO-ETEC.


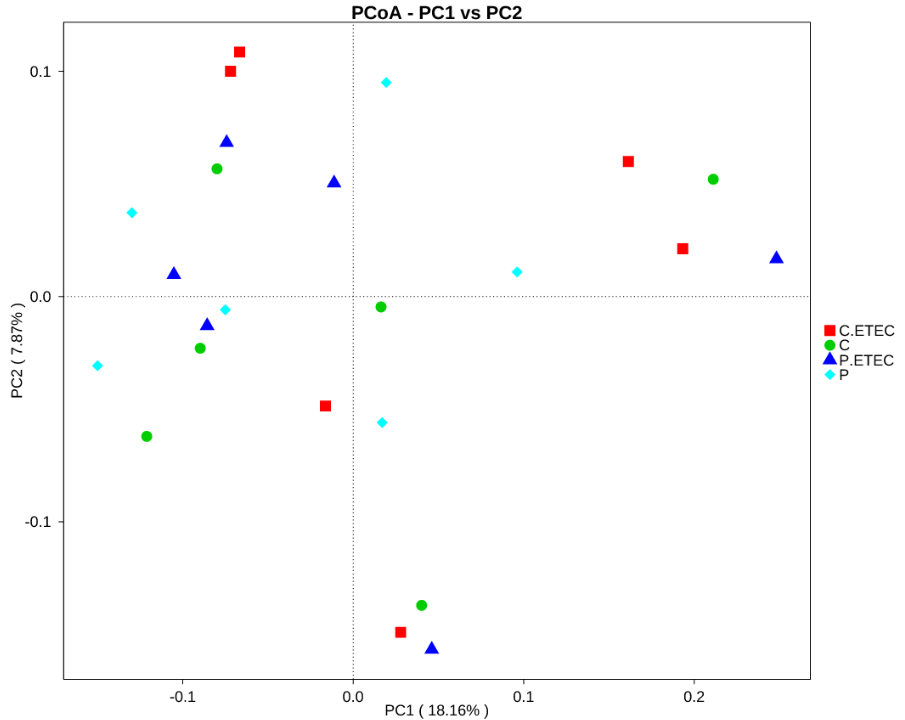

Supplement: Supplementary file 1 — Table S1. Effect of E. faecium on alpha diversity of microbial community in colonic content of piglets challenged with ETEC K88. Table S2. Effect of E. faecium on the relative abundance for the top 30 most abundant genera in the colon of piglets challenged with ETEC K88. Table S3. Effect of E. faecium on short chain fatty acid concentrations of piglets challenged with ETEC K88. Figure S1. The experimental design of the different treatments and procedures. Figure S2. Comparison of the gut microbiota composition among four groups. Principal coordinate analysis to visualize the unweighted UniFrac distances of colon digesta samples from individual piglet. (DOCX 137 kb) [file 40104_2019_376_MOESM1_ESM.docx]
